# Impact of different cryotherapy interventions on post-exercise acute delayed-onset muscle soreness, athletic performance, and inflammatory biomarkers: a systematic review and network meta-analysis

**DOI:** 10.3389/fspor.2026.1819396

**Published:** 2026-04-20

**Authors:** Jiawei Wu, Anjie Wang, Hao Hu, Hang Zhang

**Affiliations:** College of Physical Education, Anhui Polytechnic University, Wuhu, China

**Keywords:** cryotherapy, exercise performance, inflammatory factors, muscle soreness, network meta-analysis

## Abstract

**Background:**

Cryotherapy is a widely used physical recovery modality in post-exercise settings; however, systematic evidence identifying the optimal cryotherapy modality based on continuous time-course outcome assessments remains limited.

**Methods:**

To investigate the comparative effectiveness of different cryotherapy modalities—whole-body cryotherapy (WBC), cold water immersion (CWI), contrast water therapy (CWT), and local cold therapy (LCT)—on delayed-onset muscle soreness (DOMS), countermovement jump, and inflammatory biomarkers (creatine kinase, interleukin-6, and C-reactive protein) at multiple post-intervention time points (immediate, 1 h, 24 h, 48 h, and 72 h), using a systematic review and network meta-analysis approach. A systematic search was conducted in the Web of Science, PubMed, Cochrane Library, Embase, and EBSCO databases for studies published between January 1, 2010, and November 1, 2025.

**Results:**

A total of 51 randomized controlled trials comprising 1,243 participants were included. The effects of cryotherapy demonstrated a pronounced time-dependent pattern. Compared with control conditions, no cryotherapy modality significantly reduced DOMS immediately after exercise. CWI significantly attenuated DOMS at 1 h [MD = −1.09, 95% CI (−1.93, −0.24), *P* < 0.05] and 24 h [MD = −0.89, 95% CI (−1.33, −0.45), *P* < 0.05], whereas LCT produced the greatest reduction at 48 h [MD = −1.17, 95% CI (−2.19, −0.16), *P* < 0.05]. Regarding inflammatory biomarkers, WBC resulted in the largest reductions in CK levels at 48 h [MD = −118.24, 95% CI (−173.49, −63.00), *P* < 0.05] and 72 h [MD = −135.03, 95% CI (−204.40, −65.66), *P* < 0.05]. CWI was the most effective modality for suppressing IL-6 immediately post-exercise [MD = −0.32, 95% CI (−0.58, −0.06), *P* < 0.05] and at 24 h [MD = −0.46, 95% CI (−0.92, −0.01), *P* < 0.05]. In terms of exercise performance, WBC significantly improved countermovement jump performance at 1 h [MD = 9.15, 95% CI (5.12, 13.17), *P* < 0.05], 24 h [MD = 10.70, 95% CI (1.16, 20.42), *P* < 0.05], and 48 h [MD = 10.50, 95% CI (3.37, 17.62), *P* < 0.05], with sustained benefits observed up to 72 h**.**

**Conclusion:**

Cryotherapy is an effective strategy for promoting the recovery of physiological indicators following acute exercise, with its efficacy demonstrating pronounced time-dependent characteristics.

**Systematic Review Registration:**

Identifier, 2026 CRD420261300174.

## 1 Introduction

Delayed-onset muscle soreness (DOMS) is a common physiological phenomenon that often limits exercise performance. It is typically induced by unaccustomed or high-intensity eccentric exercise and peaks 24–72 h post-exercise (1). Substantial evidence indicates that DOMS is not caused by simple lactate accumulation, but rather by microstructural damage to muscle fibers induced by eccentric contractions and the subsequent inflammatory cascade. This process is characterized by increased sarcolemmal permeability, disruption of intracellular calcium homeostasis, and activation of proteolytic enzyme systems, ultimately leading to the production of pro-inflammatory markers such as creatine kinase (CK), interleukin-6 (IL-6), and C-reactive protein (CRP), and the development of localized inflammatory responses (2–8). Although various cooling methods such as CWI and WBC have been widely used as recovery strategies among elite athletes and fitness enthusiasts, their effectiveness is controversial. This controversy may be due to the following reasons: first, there are essential differences in the physiological pathways and intensities of different cooling therapies. Second, post-exercise recovery is a dynamic process, with immediate neuromuscular fatigue, early inflammatory response, and delayed muscle repair dominating the physiological state at different time stages. However, the existing reviews mostly focus on a single cooling method or combining data at different time points, and lack of a unified framework to compare the differential effects of different cooling therapies on subjective pain, objective function and biochemical inflammatory markers in key recovery time Windows such as immediately after exercise, 24 hours and 72 hours (2, 4, 5, 7). Therefore, according to the different purposes of exercise participation, it is crucial to clarify which cooling therapy method is the most effective for what indicators at what time point for optimizing the recovery strategy after acute exercise (5).

Among the many passive recovery methods after exercise, the effect of intervention measures based on hypothermia therapy such as CWI is evaluated by the subjective score of delayed muscle soreness, the composite score of perceived exertion (RPE), and the objective weakening level of phosphocreatine kinase (CK) (9). There are many potential physiological and biomechanical mechanisms for the measures of cold therapy to promote recovery. First, low temperature stimulation induces strong contraction of peripheral blood vessels and reduces local blood flow and inflammatory cell infiltration, which may reduce tissue edema and secondary injury. At the same time, low temperature can reduce nerve conduction velocity, improve pain threshold, and directly relieve pain (4). Secondly, cold exposure may also activate the sympathetic nervous system, promote the release of norepinephrine and other hormones, and participate in the regulation of inflammatory response and pain perception. Finally, by reducing pain and inflammation, cold therapy may help to maintain or restore muscle extension and joint range of motion faster, creating favorable conditions for the recovery of sports performance (6, 7). Despite their widespread application, the effectiveness of these interventions across different post-exercise time points and outcome measures remains controversial, with inconsistent findings reported across studies (3, 10–12). Post-exercise recovery is a dynamic, multifactorial, and time-dependent process, in which distinct physiological mechanisms predominate during the immediate, early (≤1 h), and delayed (24–72 h) phases following exercise (1, 10). A key limitation of the existing literature is the heterogeneity of outcome measures and assessment time points. Specifically, neuromuscular function impairments observed immediately or shortly after exercise are largely attributable to fatigue-related mechanisms, whereas muscle soreness assessed 24–72 h post-exercise is more closely associated with muscle fiber damage and inflammatory processes, exhibiting distinct temporal characteristics following exercise-induced muscle damage (1, 11, 12). However, most previous reviews and meta-analyses have synthesized outcomes at a single time point and for a single endpoint, thereby limiting the interpretability and clinical applicability of their conclusions. In summary, although numerous studies have explored the effects of cooling therapy on post-exercise recovery, there are significant limitations to the available evidence (3, 10, 11, 13, 14), to date no systematic network meta-analysis (NMA) has comprehensively compared the effects of multiple cryotherapy interventions across several post-exercise time points on subjective symptoms (DOMS), objective performance outcomes (countermovement jump, CMJ), and biochemical inflammatory markers. Most systematic reviews or meta-analyses only compare the effect of a certain cooling therapy and passive recovery, or are limited to a single time point, and fail to quantify the relative efficacy of various commonly used cooling therapies such as WBC, CWI, CWT, and LCT in a unified model by NMA. Moreover, the recovery after exercise is sequential. Pooling data from different time points may obscure the role of cooling in specific critical recovery Windows.

**Figure 1:**
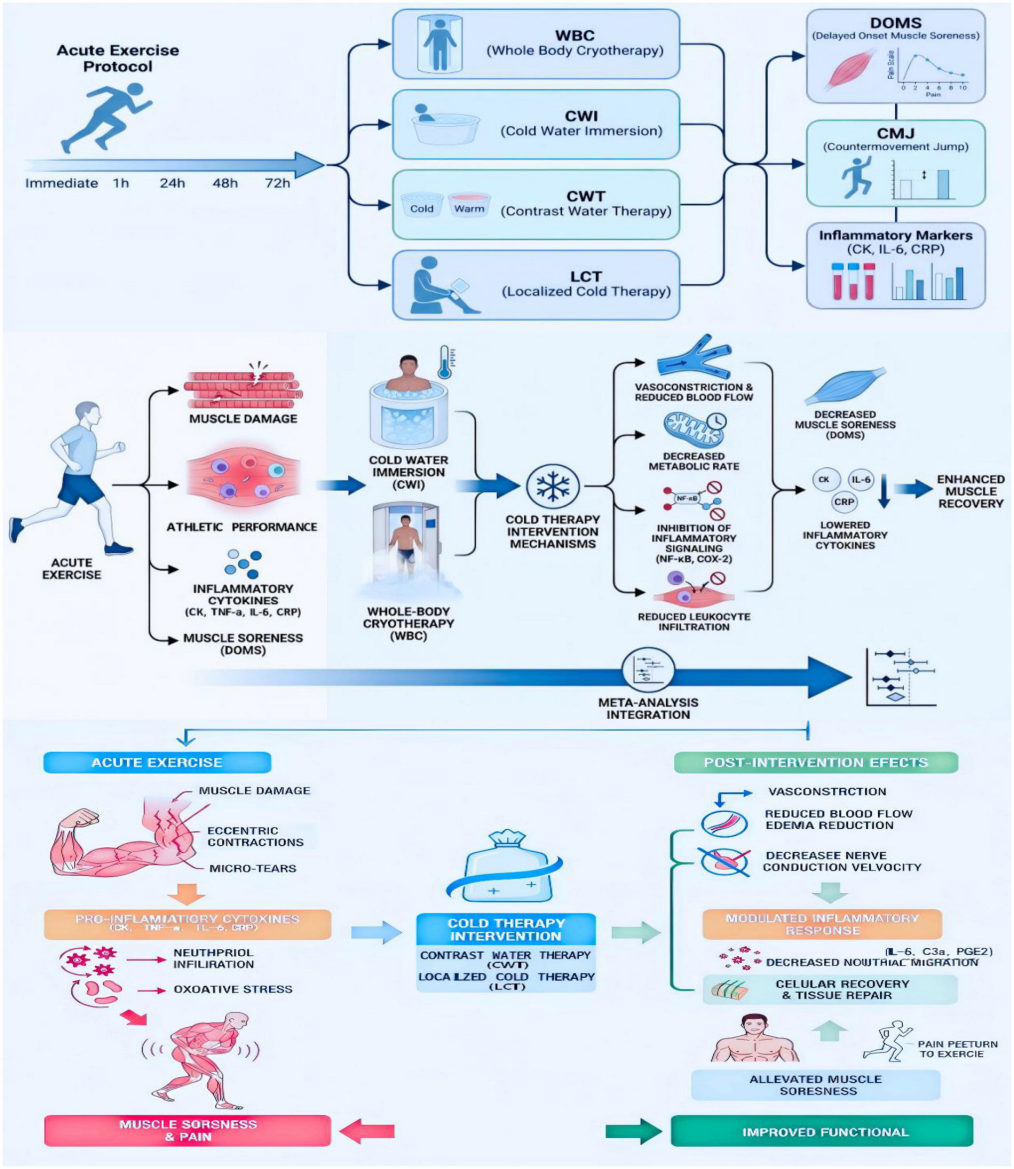
Schematic flow diagram illustrating the mechanistic pathways by which different cryotherapy interventions influence delayed-onset muscle soreness, exercise performance, and inflammatory markers following acute exercise. CK, creatine kinase; IL-6, interleukin-6; CRP, C reactive protein; TNF-*α*, tumor necrosis factor; *α*PGE2, prostaglandin E2.

Therefore, the present study aimed to conduct a systematic review and network meta-analysis to compare the effects of four commonly used cryotherapy interventions (WBC, CWI, CWT, and LCT) on recovery from delayed-onset muscle soreness (DOMS), countermovement jump (CMJ) performance, and inflammatory markers (CK, IL-6, and CRP) at multiple post-exercise time points (immediately, 1 h, 24 h, 48 h, and 72 h), and to identify the optimal cryotherapy strategy (Figure 1). By integrating time-resolved analyses with comparative intervention evaluation, this study seeks to provide evidence-based and practical guidance for alleviating exercise-induced fatigue in recreational exercisers and for helping elite athletes maintain optimal performance during training and competition across the competitive season.Based on the diversity of cold therapy mechanisms and the sequential characteristics of injury and recovery process after exercise, we speculate that CWI may be more effective in relieving pain and local inflammation in the early stage after exercise, while WBC may be more advantageous in reducing inflammatory factors and improving exercise performance in the late stage of recovery. CWT and LCT may have different patterns of influence on DOMS, inflammatory factors, and exercise performance at a single time point.

## 2 Methods

### 2.1 Study design

This systematic review was conducted in accordance with the Cochrane Handbook for Systematic Reviews of Interventions (67) and reported following the Preferred Reporting Items for Systematic Reviews and Meta-Analyses (PRISMA) checklist (67) (Appendix Table 1). The review protocol was registered in PROSPERO (registration number: 2026 CRD420261300174).

### 2.2 Literature search strategy

A comprehensive electronic literature search was performed in MEDLINE (PubMed), Embase, Cochrane Library, EBSCO, and Web of Science. The search strategy was developed based on Medical Subject Headings (MeSH) terms and keywords structured according to the PICOS framework: (P) population—acute exercise, athletes, exercisers, healthy adults; (I) intervention—cryotherapy, cold-water immersion, whole-body cryotherapy, localized cryotherapy; (C) comparator—non-cryotherapy interventions, passive recovery; (O) outcomes—delayed-onset muscle soreness, exercise performance, inflammatory markers; (S) study design—randomized controlled trials. The search covered studies published between January 1, 2010, and November 1, 2025. Only articles published in English were considered. In addition, reference lists of relevant studies were manually screened to identify further eligible publications. The detailed search strategy is presented in Appendix Figure 1.

### 2.3 Eligibility criteria

Muscle soreness and fatigue are commonly assessed using subjective scales and are typically manifested as delayed-onset muscle soreness (DOMS) and ratings of perceived exertion (RPE) (3). The countermovement jump (CMJ) is considered a practical and valuable indicator of muscle strength and athletic performance, serving as an important predictor of maximal velocity and explosive power, and as an effective method for assessing functional capacity in fatigued athletes (68). Plasma concentrations of creatine kinase (CK), interleukin-6 (IL-6), and C-reactive protein (CRP) are widely used as indirect biochemical markers of exercise-induced muscle damage, reflecting the extent of muscle membrane disruption (69). IL-6 is traditionally regarded as a marker of post-exercise inflammation, while CRP serves as an indicator of infection or inflammatory status (14). Accordingly, studies were included if they met the following criteria: (1) peer-reviewed controlled trials; (2) adult participants without known clinical diseases or musculoskeletal injuries; (3) interventions involving WBC, CWI, CWT, or LCT compared with a control condition; (4) reporting at least one of the following outcomes: DOMS, CMJ, CK, IL-6, or CRP; and (5) outcome measurements conducted at matched post-exercise time points, including immediate, 1 h, 24 h, 48 h, or 72 h.

### 2.4 Study characteristics and data extraction

Two authors independently screened titles and abstracts of potentially eligible studies and subsequently reviewed the full texts to determine eligibility. Discrepancies were resolved through discussion with a third reviewer. When necessary, corresponding authors were contacted to obtain missing data. EndNote X9 (Clarivate Analytics, Philadelphia, PA) was used to manage references and remove duplicates. Extracted data included: (1) basic study characteristics (title, first author, publication year); (2) participant characteristics (sex, sample size); (3) intervention details (WBC, CWI, CWT, LCT); (4) outcome measures (DOMS, CMJ, CK, IL-6, CRP); and (5) timing of outcome assessments (immediate, 1 h, 24 h, 48 h, and 72 h). The main characteristics of the included studies are summarized in Appendix Table 2.

### 2.5 Risk of bias assessment

The risk of bias for each included randomized controlled trial was independently assessed by two authors using the Cochrane Risk of Bias tool for RCTs as outlined in the Cochrane Handbook for Systematic Reviews of Interventions (Version 7.2.0) (15–17). The following domains were evaluated: random sequence generation, allocation concealment, blinding of outcome assessors, completeness of outcome data, selective reporting, and other potential sources of bias. Based on the number of domains judged as having a high, unclear, or low risk of bias, studies were categorized as having a high, moderate, or low overall risk of bias.

### 2.6 Data synthesis and statistical analysis

Network meta-analyses (NMA) were performed using Stata version 17.0, with outcome measures from the included studies treated as pooled continuous variables (18). When outcome units were consistent across studies, mean differences (MDs) were used as the effect size; when units differed, values were converted using an international physiological unit conversion tool (UnitsLab.com). A random-effects multivariate NMA was conducted within a frequentist framework. Network geometry was illustrated using network plots, in which nodes represent interventions (with node size proportional to the total sample size allocated to each cryotherapy modality) and edges indicate direct comparisons between two interventions, with edge thickness corresponding to the number of studies contributing to each comparison (19). Global consistency was first assessed using loop inconsistency tests. This was followed by inconsistency model testing and local inconsistency assessment using the node-splitting method. When no significant inconsistency was detected (*P* > 0.05), a consistency model was applied. Local inconsistency analyses included node-splitting and comparisons between direct and indirect estimates (20). Heterogeneity was quantified by reporting the between-study variance (*τ*) for all pairwise comparisons. In the presence of substantial heterogeneity, subgroup analyses and sensitivity analyses were conducted, or meta-analyses were repeated after excluding studies with outlying results. Given the considerable variability in acute exercise protocols, participant characteristics, and intervention modalities, studies with a high risk of bias were excluded, and leave-one-out sensitivity analyses were further performed to assess the robustness of the findings (21). Publication bias was examined using funnel plots. The overall statistical significance of treatment effects was evaluated based on the fitted inconsistency model. Finally, interventions were ranked according to the surface under the cumulative ranking curve (SUCRA), with higher values indicating greater effectiveness of the intervention (22).

## 3 Results

### 3.1 Literature search results

**Figure 2:**
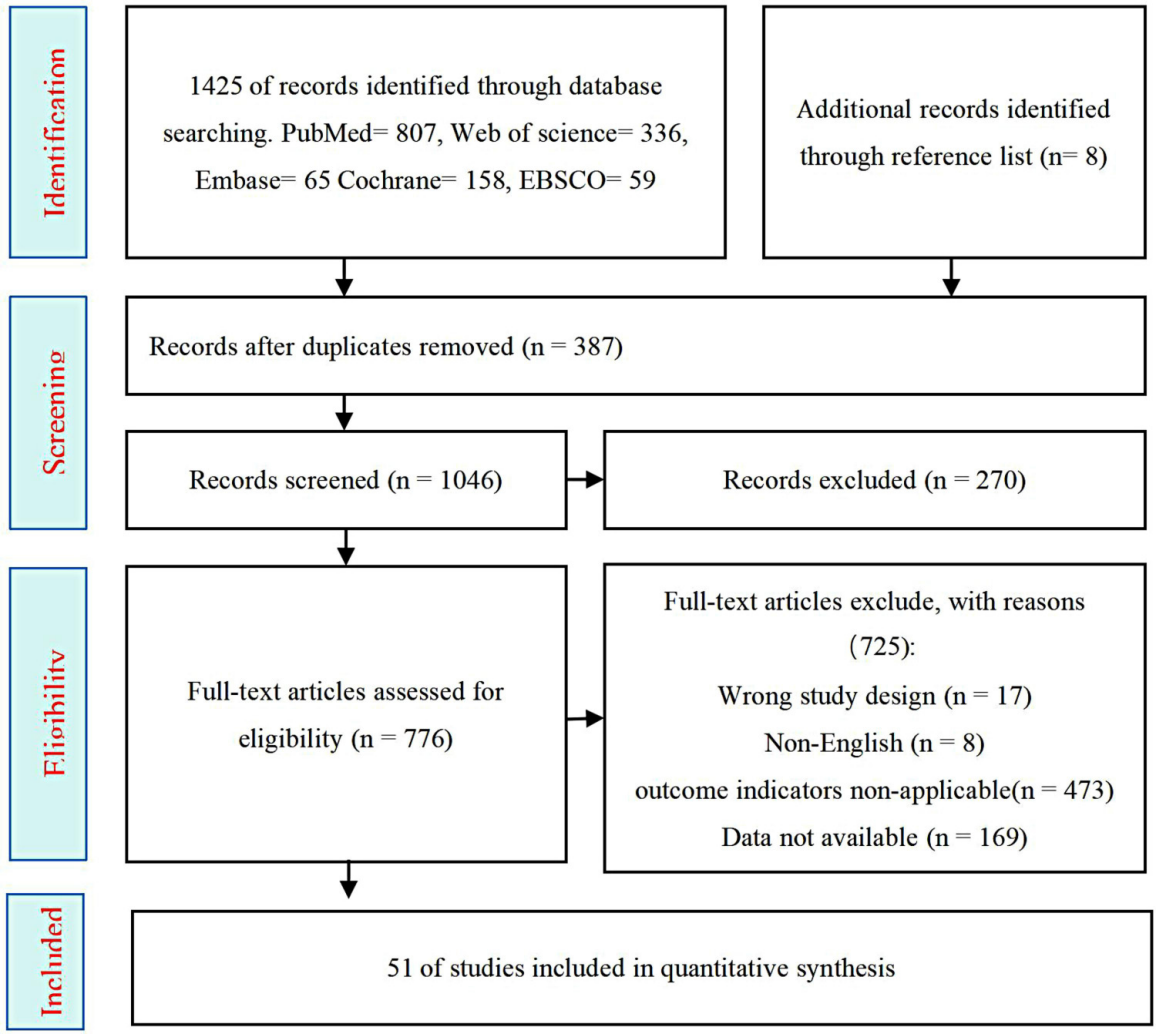
Flowchart of the preferred reporting items for systematic reviews and meta-analyses.

A total of 1,433 records were retrieved through database searches: PubMed (*n* = 807), Embase (*n* = 65), Cochrane Library (*n* = 158), Web of Science (*n* = 336), and EBSCO (*n* = 59). An additional 8 records were identified through other sources. After removing 387 duplicates, 725 records were excluded based on title and abstract screening for not meeting inclusion criteria. Full-text assessment of 473 articles led to further exclusions due to ineligible outcome measures, including 17 studies with inappropriate study designs, 169 studies with data that could not be extracted, and 83 studies with irrelevant topics. Ultimately, 51 randomized controlled trials were included in the analysis (Figure 2).

### 3.2 Characteristics of included studies

The basic characteristics of the included studies are summarized in Appendix Table 2. Most studies were published within the last 15 years. Outcome reporting included creatine kinase (CK) in 44 studies (7, 21, 23–44, 63–65, 70–78), interleukin-6 (IL-6) in 12 studies (24, 27, 29, 34, 45–47, 66, 71, 72, 77, 78), C-reactive protein (CRP) in 17 studies (24, 28–30, 35, 41, 43, 45, 47–52, 71, 72, 74, 77) delayed-onset muscle soreness (DOMS) in 24 studies (23, 24, 27, 29, 31, 34, 36–38, 43, 49, 53–58, 71, 72, 73, 74, 76, 79, 80), and countermovement jump (CMJ) in 24 studies (23, 24, 27, 29, 31, 34, 36–38, 43, 49, 55–58, 64, 71, 72, 73, 74, 76, 79, 80).

### 3.3 Risk of bias assessment of included studies

The results of the risk of bias assessment are presented in Appendix Figures 2, 3. Four studies did not clearly describe the method of group allocation (44, 47, 71, 78) while two studies used non-random allocation, indicating high risk (31, 53). Twenty-one studies did not report allocation concealment, representing high risk (25, 26, 28, 34, 37, 59, 70, 72, 77). For the completeness of outcome data, studies with >20% loss to follow-up without intention-to-treat analysis would be rated as high risk; none of the included studies exceeded this threshold. In the domain of other biases, factors such as lack of supervision of exercise interventions or potential conflicts of interest were rated as high risk. Overall study quality was classified according to established criteria: studies with no high-risk domains and ≤3 unclear-risk domains were rated as high quality; studies with one high-risk domain or no high-risk domains but ≥4 unclear-risk domains were rated as moderate quality; all other studies were rated as low quality. Accordingly, 37 studies were rated as high quality, 12 as moderate quality (25, 26, 28, 34, 37, 50, 53, 59, 70, 72, 74, 77), and 2 as low quality (37, 41).

### 3.4 The results of the network meta-analysis

#### 3.4.1 Reporting of conventional meta-analysis results

Because analyses were stratified by outcome measure, the number of studies available for certain endpoints—specifically IL-6 at 72 h and CRP at 72 h following cryotherapy—was insufficient to perform a network meta-analysis. Therefore, conventional pairwise meta-analyses were conducted for these outcomes. Heterogeneity among studies was assessed using the I^2^ statistic and corresponding P-values, with P-values indicating statistical significance. Pooled effect sizes were reported as mean differences (MD) with 95% confidence intervals (MD, 95% CI).

#### 3.4.2 Reporting of network meta-analysis results

Network meta-analysis (NMA) results are presented according to network geometry, consistency assessment, publication bias, pooled effect estimates, and SUCRA-based rankings. In consistency model testing, *P* < 0.05 was considered statistically significant. SUCRA rankings were generated by combining both statistically significant and non-significant results, with only the ranking order reported.The evidence network in this study showed unbalanced structural features. CWI and CWT form the core of the network and have the largest number of direct comparative studies, while LCT is linked to other interventions mainly through indirect comparisons. This structure means that most comparisons involving LCT lack research evidence for direct comparisons and rely solely on indirect estimates through common controls. Although we did not detect significant statistical inconsistencies by loop inconsistency test, inconsistency model and node segmentation (*P* > 0.05), this study pointed out that the power of these statistical tests is limited in comparisons where evidence is sparse, especially in those lacking closed-loop structure such as LCT. Therefore, the uncertainty of comparative results based on indirect evidence is relatively high and should be interpreted with extra caution.

#### 3.4.3 Secondary outcome

##### 3.4.3.1 Ck

**Figure 3:**
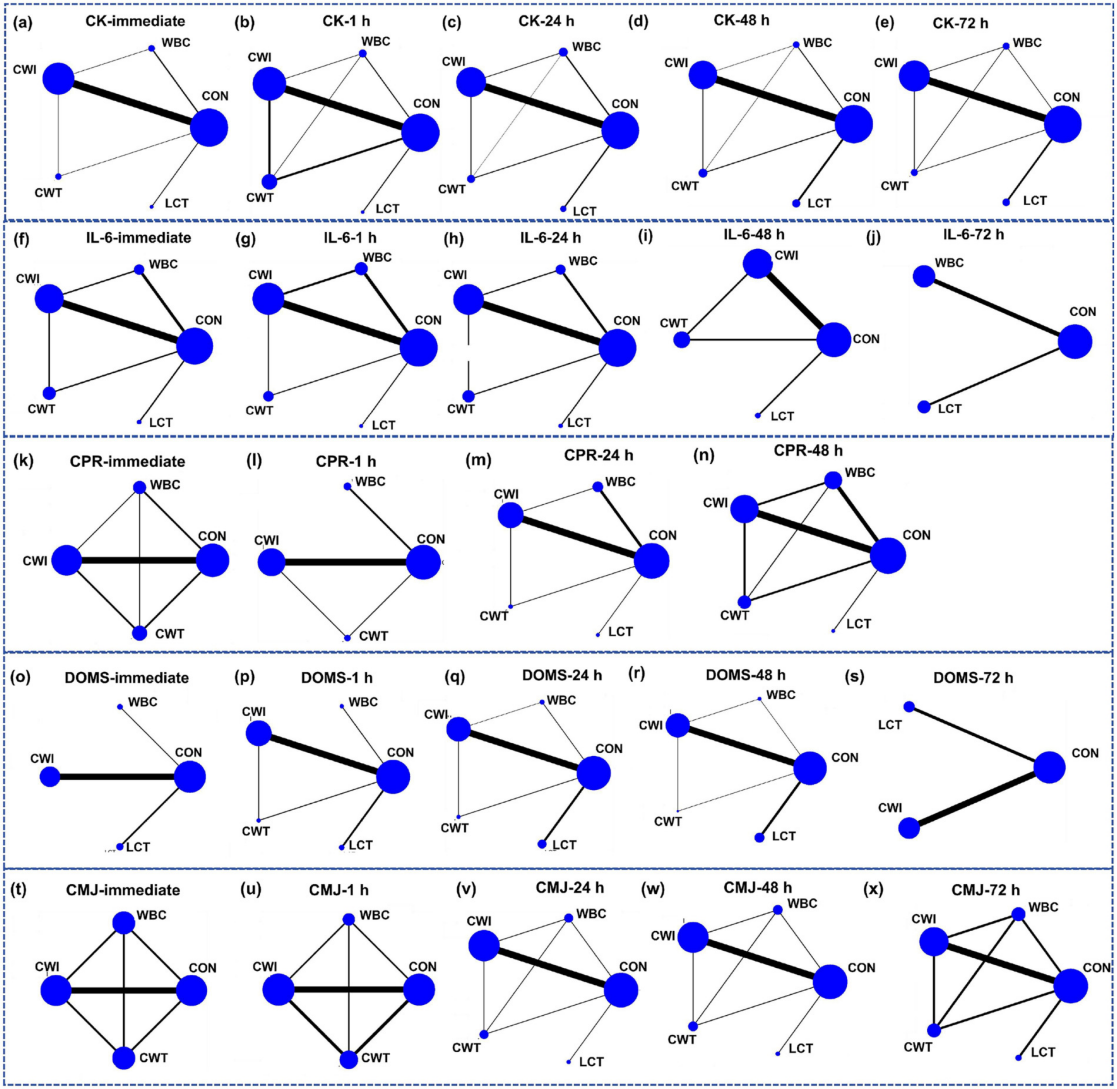
Network evidence diagram illustrating the effects of different cold therapy interventions on acute post-exercise delayed muscle soreness, exercise performance, and inflammatory factors. CON, control; WBC, whole-body cryotherapy; CWI, cold water immersion; CWT, cold water contrast; LCT, local cold therapy; DOMS, delayed-onset muscle soreness; CMJ, countermovement jump; CK, creatine kinase; IL-6, interleukin-6; CRP, C reactive protein, **(a-e)**; CK-Multi-period Network Evidence Graph, **(f-j)**; IL-6-Multi-period Network Evidence Graph, **(k-n)**, CRP-Multi-period Network Evidence Graph, **(o-s)**, DOMS-Multi-period Network Evidence Graph, **(t-x)**; CMJ-Multi-period Network Evidence Graph.

The network geometry for CK is shown in Figure 3(a–e). Cold-water immersion (CWI) was the most frequently applied intervention, while localized cryotherapy (LCT) was the least frequently used. Except for the comparisons CWI vs WBC, CWI vs CWT, and WBC vs CWT, which had mixed direct and indirect evidence, the majority of CK comparisons across different time points were based on indirect evidence. Consistency of the network was assessed using loop inconsistency tests, consistency models, and node-splitting methods. Loop inconsistency tests indicated that all triangular loops involving WBC and CWI were consistent (*P* > 0.05). LCT was compared only indirectly with other interventions and did not form closed loops. Other loops showed good consistency. Inconsistency model testing revealed P-values > 0.05 for all outcomes, indicating no significant inconsistency, supporting the use of the consistency model. Node segmentation method showed that no significant statistical inconsistency was found between direct comparison and indirect comparison of each outcome index within the existing evidence network (*P* > 0.05). However, considering the sparse evidence of LCT and the lack of direct comparison, this result is not absolutely reliable. Therefore, CK result analysis should be interpreted with caution in combination with network structure characteristics. Publication bias was assessed via funnel plots, which demonstrated good symmetry for all outcomes, suggesting minimal influence of publication bias or small-study effects (Appendix’s Table 4 and Figures 5, 6).

##### 3.4.3.2 Pooled effect sizes and sucra rankings

**Figure 4:**
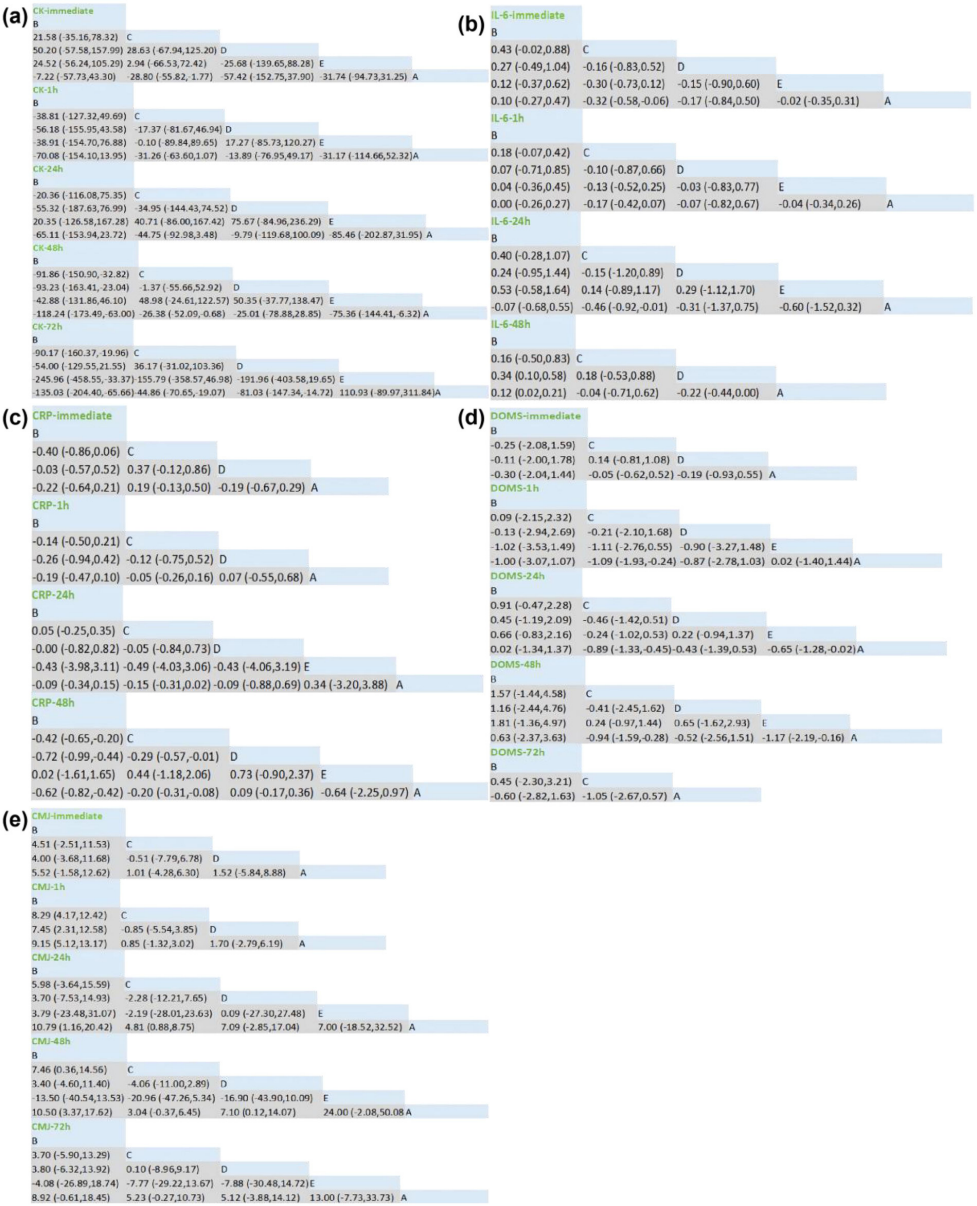
Results of the network meta-analysis. A, control; B, whole-body cryotherapy; C, cold water immersion; D, cold water contrast; E, local cold therapy; DOMS, delayed-onset muscle soreness; CMJ, countermovement jump; CK, creatine kinase; IL-6, interleukin-6; CRP, C reactive protein, **(a)**: CK - Multi- Period Inverted Triangle Result, **(b)**: IL-6 - Multi- Period Inverted Triangle Result, **(c)**: CRP - Multi- Period Inverted Triangle Result, **(d)**: DOMS - Multi- Period Inverted Triangle Result, **(e)**: CMJ - Multi- Period Inverted Triangle Result.

**Figure 5:**
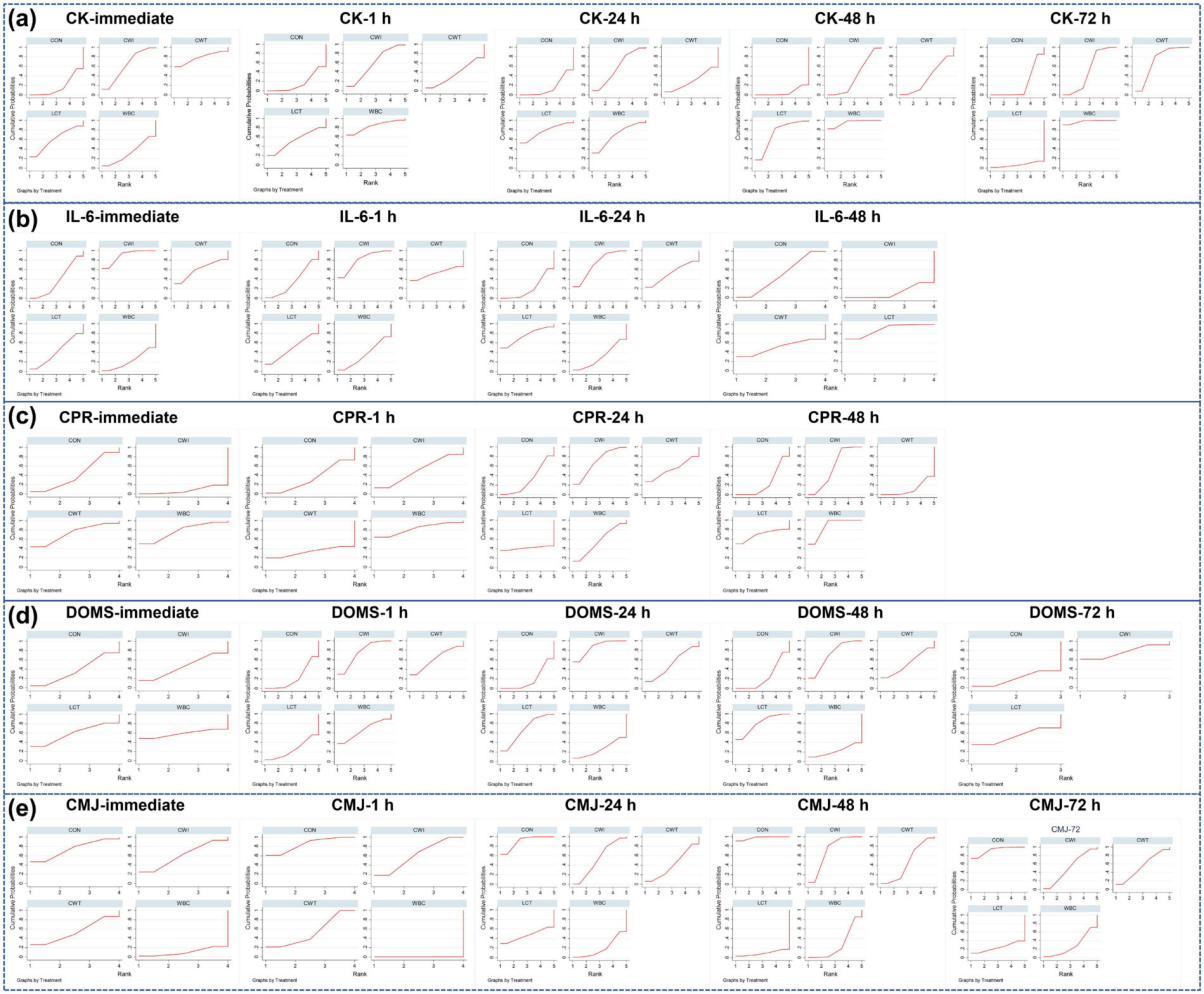
Cumulative probability ranking of sUCRA. CON, control; WBC, whole-body cryotherapy; CWI, cold water immersion; CWT, cold water contrast; LCT, local cold therapy; DOMS, delayed-onset muscle soreness; CMJ, countermovement jump; CK, creatine kinase; IL-6, interleukin-6; CRP, C reactive protein, **(a)**: CK Multi-period SUCRA Ranking, **(b)**: IL-6 Multi-period SUCRA Ranking, **(c)**: CRP Multi-period SUCRA Ranking, **(d)**: DOMS Multi-period SUCRA Ranking, **(e)**: CMJ Multi-period SUCRA Ranking.

Overall, different cryotherapy modalities exhibited varying degrees of effectiveness in reducing CK compared with control across multiple time points. Further analysis showed that although the immediate, 1 h, and 24 h CWT [MD = 57.42, 95% (152.75, 37.90), *P* > 0.05), the WBC [MD = 70.08, 95% (154.10, 13.95), *P* > 0.1), the LCT [MD = 85.46, 95%(-202.87,31.95), *P* > 0.05] had a certain effect on reducing CK, but the result was not significant. Only CWI in immediate: [MD = −28.80, 95% (−55.82,-1.77), *P* < 0.05] was significant and statistically significant. CWI at 1 h [MD = −31.26, 95%(-63.60,1.07), *P* = 0.058] and 24 h[MD = −44.75,95%(-92.98,3.48), *P* = 0.069] had a decreasing trend in reducing CK effect, but did not reach statistical significance. CWT at 48 h [MD = −25.01, 95%(-78.88,28.85), *P* > 0.1] and LCT at 72 h [MD = −110.93, 95%(-89.97,311.84), *P* > 0.1] had some effect, but the results were not significant and statistically significant. The remaining three intervention methods had a certain effect on reducing CK, and the results were significant. SUCRA rankings indicated the relative probability of being the most effective intervention at each time point: (Figures 4a, 5a)

- Immediate: CWT highest (SUCRA = 78), followed by CWI (SUCRA = 62.4) and WBC (SUCRA = 60.4), with control (CON) lowest (SUCRA = 17.2).
- 1 h: WBC highest (SUCRA = 83.9), followed by CWI (SUCRA = 59.6) and LCT (SUCRA = 53.4), CON lowest (SUCRA = 16.5).
- 24 h: LCT highest (SUCRA = 77.1), followed by WBC (SUCRA = 69.7) and CWI (SUCRA = 57.9), CON lowest (SUCRA = 15.7).
- 48 h: WBC highest (SUCRA = 95.4), followed by LCT (SUCRA = 73.3) and CWI (SUCRA = 39.8), CON lowest (SUCRA = 5.6).
- 72 h: WBC highest (SUCRA = 97.5), followed by CWT (SUCRA = 72) and CWI (SUCRA = 52), with LCT lowest (SUCRA = 6.9).

These results indicate that WBC demonstrates the most consistent and pronounced efficacy in reducing CK at later time points (48–72 h), whereas CWI, CWT, and LCT show time-dependent effects that vary across earlier post-exercise intervals.

##### 3.4.3.3 IL-6

**Figure 6:**
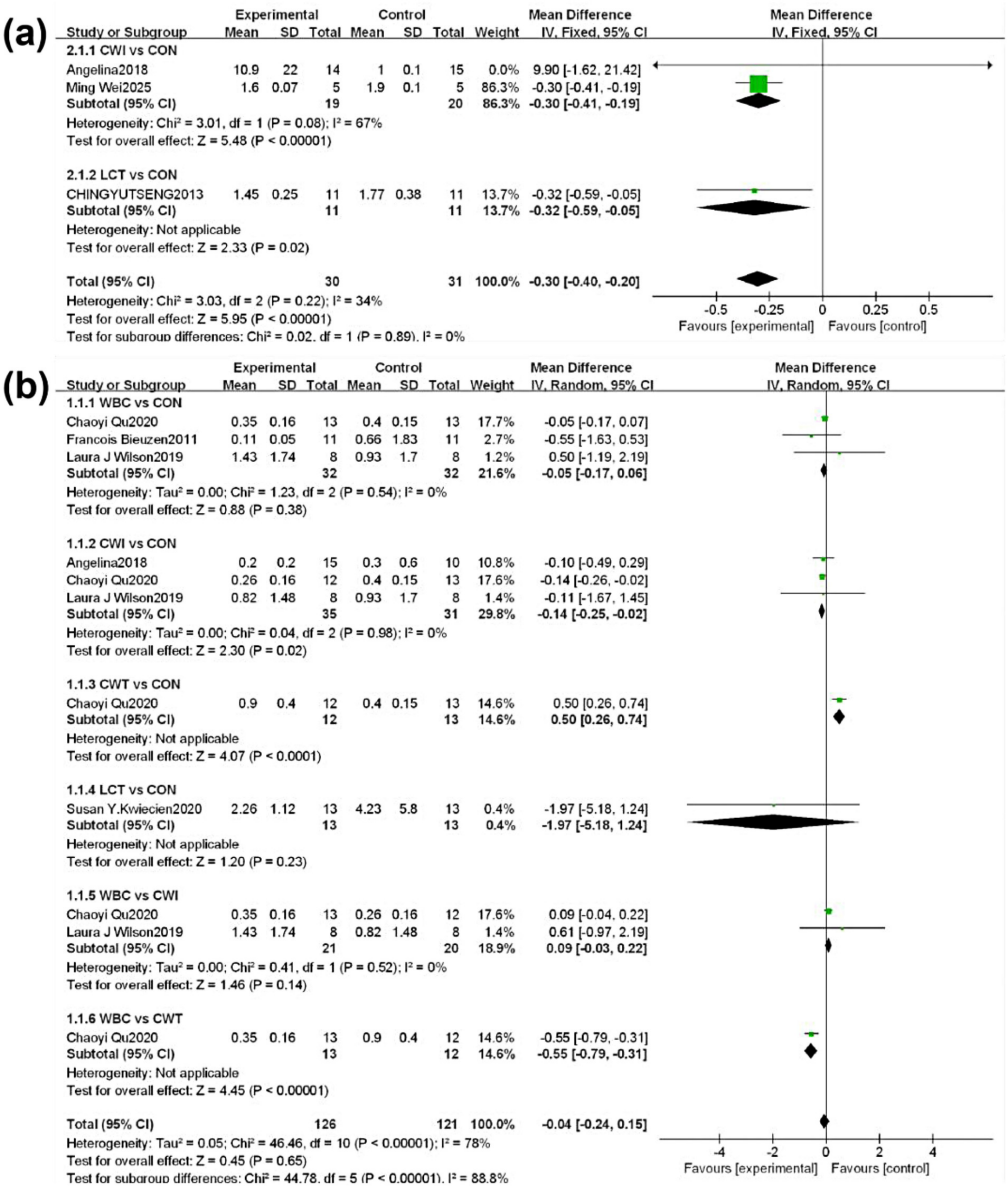
Results of the conventional meta-analysis. **(a)**: The results of the 72-hour regular meta-analysis of IL-6, **(b)**: The results of the 72-hour regular meta-analysis of CRP.

The network geometry for IL-6 is shown in Figure 3(f–j). Cold-water immersion (CWI) was the most frequently applied intervention, while localized cryotherapy (LCT) was the least frequently used. Except for the comparisons CWI vs WBC and CWI vs CWT, which included mixed direct and indirect evidence, most IL-6 comparisons across multiple time points were based on indirect evidence. At 48 h, no WBC intervention arm was available, leaving only CWT vs CWI as mixed evidence. At 72 h, due to the limited number of studies, network meta-analysis was not feasible, and conventional pairwise meta-analysis was applied. Inconsistency of the network was evaluated using loop inconsistency tests, consistency models, and node-splitting analyses. Loop inconsistency tests indicated that all triangular loops involving WBC, CWI, and CWT were consistent (*P* > 0.05). LCT was only compared indirectly with other interventions and did not form closed loops, while other loops demonstrated good consistency. Inconsistency model testing revealed P-values > 0.05 for all outcomes, indicating no significant inconsistency and supporting the use of the consistency model. Node segmentation method showed that no significant statistical inconsistency was found between direct comparison and indirect comparison of each outcome index within the existing evidence network (*P* > 0.05). However, considering the sparse evidence of LCT and the lack of direct comparison, this result is not absolutely reliable. Therefore, the analysis of IL-6 results should be interpreted with caution in combination with the characteristics of network structure. Publication bias was assessed using funnel plots, which demonstrated good symmetry for all IL-6 outcomes, suggesting minimal influence from publication bias or small-study effects (Appendix’s Table 4 and Figures 5, 6).

##### 3.4.3.4 Pooled effect estimates and sucra rankings

Overall, different cryotherapy modalities exhibited varying degrees of effectiveness in reducing IL-6 compared with control across multiple post-exercise time points. Detailed analysis showed the following: (Figures 4b, 6a, 6b)

- Immediate: CWT (MD = −0.17, 95% CI (−0.84, 0.50), *P* > 0.05) and WBC (MD = 0.10, 95% CI (−0.27, 0.47), *P* > 0.05) showed some effect, but these were not statistically significant. Only CWI (MD = −0.32, 95% CI (−0.58, −0.06), *P* < 0.05) demonstrated a significant reduction in IL-6.
- 1 h: All four cryotherapy modalities showed some reduction in IL-6, but none reached statistical significance.
- 24 h: CWI (MD = −0.46, 95% CI (−0.92, −0.01), *P* < 0.05) demonstrated a significant reduction in IL-6 compared with other cryotherapy modalities.
- 48 h: LCT[MD = −0.22,95% (−0.44,0.00), *P* = 0.053] tended to decrease the effect of IL-6 reduction, but did not reach statistical significance.
- 72 h: CWT (MD = −0.30, 95% CI (−0.41, −0.19), *P* < 0.000001) significantly reduced IL-6 compared with control. LCT (MD = −0.32, 95% CI (−0.59, −0.05), *P* < 0.05) also demonstrated significant reduction compared with control.

SUCRA Rankings indicated the probability of each intervention being the most effective at each time point:

- Immediate: CWI highest (SUCRA = 89.2), followed by CWT (SUCRA = 60.6) and LCT (SUCRA = 41.1), with control lowest (SUCRA = 37).
- 1 h: CWI highest (SUCRA = 79.9), followed by CWT (SUCRA = 52.7) and LCT (SUCRA = 47.7), control lowest (SUCRA = 34.7).
- 24 h: LCT highest (SUCRA = 74.8), followed by CWI (SUCRA = 71.7) and CWT (SUCRA = 52.7), control lowest (SUCRA = 20.5).
- 48 h: CWT highest (SUCRA = 88.4), followed by CWI (SUCRA = 51.8) and control (SUCRA = 48.9), with WBC lowest (SUCRA = 10.9).

These findings suggest that CWI is most effective in the early post-exercise phase (immediate to 1 h), whereas CWT and LCT demonstrate greater efficacy in the later recovery phase (48–72 h).

##### 3.4.3.5 Crp

The network geometry for CRP is shown in Figure 3(k–n). Cold-water immersion (CWI) and whole-body cryotherapy (WBC) were the most frequently applied interventions, while localized cryotherapy (LCT) was used least often. At the immediate time point, no LCT intervention arms were available, and all comparisons were based on mixed evidence. At 1 h, there was no LCT arm; CWI, CWT, and control (CON) were based on mixed evidence, while other comparisons were indirect. At 24 h, only CWI vs WBC and CWI vs CWT involved mixed evidence, with all other comparisons based on indirect evidence. At 48 h, all comparisons except LCT were based on mixed evidence, with LCT only indirectly compared. At 72 h, due to limited study numbers, network meta-analysis was not feasible, and conventional meta-analysis was applied. Network inconsistency was assessed using loop inconsistency tests, consistency models, and node-splitting analyses. Loop inconsistency tests indicated that all triangular loops involving WBC, CWI, and CWT were consistent (*P* > 0.05). LCT comparisons were only indirect and did not form closed loops. Other loops showed good consistency. Inconsistency model testing yielded *P* > 0.05 for all outcomes, indicating no significant inconsistency and supporting the use of the consistency model. Node segmentation method showed that no significant statistical inconsistency was found between direct comparison and indirect comparison of each outcome index within the existing evidence network (*P* > 0.05). However, considering the sparse evidence of LCT and the lack of direct comparison, this result is not absolutely reliable. Therefore, CPR result analysis should be interpreted with caution in combination with network structure characteristics. Publication bias assessment using funnel plots demonstrated good symmetry for all CRP outcomes, suggesting minimal influence of publication bias or small-study effects (Appendix’s Table 4 and Figures 5, 6).

##### 3.4.3.6 Pooled effect estimates and sucra rankings

Overall, different cryotherapy modalities exhibited variable effects on CRP reduction compared with control across time points. Detailed analysis revealed: (Figures 4c, 6b, 5c)

- Immediate: WBC (MD = −0.22, 95% CI (−0.64, 0.21), *P* > 0.05), CWI (MD = 0.19, 95% CI (−0.13, 0.50), *P* > 0.05), and CWT (MD = −0.19, 95% CI (−0.67, 0.29), *P* > 0.05) showed some reduction in CRP, but effects were not statistically significant.
- 1 h: WBC (MD = −0.19, 95% CI (−0.47, 0.10), *P* > 0.05), CWI (MD = −0.05, 95% CI (−0.26, 0.16), *P* > 0.05), and CWT (MD = 0.07, 95% CI (−0.55, 0.68), *P* > 0.05) similarly demonstrated some effect, without statistical significance.
- 48 h: Both WBC (MD = −0.62, 95% CI (−0.82, −0.42), *P* < 0.05) and CWI (MD = −0.20, 95% CI (−0.31, −0.08), *P* < 0.05) significantly reduced CRP, with WBC showing superior efficacy.
- 72 h: WBC (MD = −0.05, 95% CI (−0.17, 0.06), *P* = 0.38) was not significant compared with control, while CWI (MD = −0.14, 95% CI (−0.25, −0.02), *P* < 0.05) showed significant reduction. LCT (MD = −1.97, 95% CI (−5.18, 1.24), *P* > 0.05) did not significantly reduce CRP. WBC also showed a significant advantage over CWT (MD = −0.55, 95% CI (−0.79, −0.31), *P* < 0.05).

SUCRA Rankings for CRP indicated the probability of each intervention being most effective at each time point:

- Immediate: WBC highest (SUCRA = 77.9), followed by CWT (SUCRA = 73.1) and CON (SUCRA = 41.4), with CWI lowest (SUCRA = 7.6).
- 1 h: WBC highest (SUCRA = 82.9), followed by CWI (SUCRA = 50.5) and CON (SUCRA = 33.7), with CWT lowest (SUCRA = 33.0).
- 24 h: CWI highest (SUCRA = 68.7), followed by WBC (SUCRA = 55.4) and CWT (SUCRA = 53.2), with CON lowest (SUCRA = 31.0).
- 48 h: WBC highest (SUCRA = 87.4), followed by LCT (SUCRA = 69.6) and CWI (SUCRA = 57.0), with CWT lowest (SUCRA = 11.5).

These results suggest that WBC provides the most consistent and pronounced reduction in CRP at later time points, whereas CWI and other modalities have time-dependent effects that vary across the recovery period.

#### 3.4.4 Primary outcome

##### 3.4.4.1 Doms

The network geometry for DOMS is shown in Figure 3(o–s). Cold-water immersion (CWI) was the most frequently applied intervention, while whole-body cryotherapy (WBC) was applied least often. At the immediate time point, no CWT intervention arm was available, and all comparisons were based on indirect evidence. At 1 h, CWI, CWT, and control (CON) were based on mixed evidence, while other comparisons were indirect. At 24 h and 48 h, only CWT vs WBC vs CON and CWI vs CWT vs CON involved mixed evidence, with all other comparisons being indirect. At 72 h, due to the limited number of studies, all comparisons were indirect. Network consistency was evaluated using loop inconsistency tests, consistency models, and node-splitting analyses. Loop inconsistency tests indicated that for the immediate and 72 h time points there were no closed loops; for other time points, all triangular loops involving CWI, CWT, and CON were consistent (*P* > 0.05). LCT comparisons were only indirect and did not form closed loops. Other loops demonstrated good consistency. Inconsistency model testing yielded P-values > 0.05 for all outcomes, indicating no significant inconsistency and supporting the use of the consistency model.Node segmentation showed that there was no statistically significant difference between direct comparison and indirect comparison in the existing evidence network (*P* > 0.05). However, considering the sparse evidence of LCT and the lack of direct comparison, this result is not absolutely reliable. Therefore, the DOMS results should be interpreted with caution in combination with the characteristics of network structure. Funnel plot assessments indicated good symmetry, suggesting minimal influence of publication bias or small-study effects (Appendix’s Table 4 and Figures 5, 6).

##### 3.4.4.2 Pooled effect estimates and sucra rankings

Overall, different cryotherapy modalities showed varying effectiveness in reducing DOMS compared with control at different post-exercise time points. Detailed analysis revealed the following: (Figures 4d, 5d)

- Immediate and 72 h: WBC (MD = −0.30, 95% CI (−2.04, 1.44), *P* > 0.05), CWI (MD = −0.05, 95% CI (−0.62, 0.52), *P* > 0.05), CWT (MD = −0.19, 95% CI (−0.93, 0.55), *P* > 0.05), and LCT (MD = −0.60, 95% CI (−2.82, 1.63), *P* > 0.05) showed some effect on DOMS reduction, but none were statistically significant.
- 1 h: CWI (MD = −1.09, 95% CI (−1.93, −0.24), *P* < 0.05) was the most effective, with statistically significant reduction in DOMS.
- 24 h: CWI (MD = −0.89, 95% CI (−1.33, −0.45), *P* < 0.05) and LCT (MD = −0.65, 95% CI (−1.28, −0.02), *P* < 0.05) both showed significant effects, with CWI outperforming LCT.
- 48 h: LCT (MD = −1.17, 95% CI (−2.19, −0.16), *P* < 0.05) and CWI (MD = −0.94, 95% CI (−1.59, −0.28), *P* < 0.05) significantly reduced DOMS, with LCT showing greater effect than CWI.

SUCRA Rankings indicated the probability of each intervention being the most effective at each time point:

- Immediate: WBC highest, followed by LCT and CWI, with CON lowest.
- 1 h: CWI highest (SUCRA = 75.1), followed by WBC (SUCRA = 65.9) and CWT (SUCRA = 61.9), with CON lowest (SUCRA = 21.7).
- 24 h: CWI highest (SUCRA = 86.4), followed by LCT (SUCRA = 67.8) and CWT (SUCRA = 51.3), with CON lowest (SUCRA = 18.4).
- 48 h: LCT highest (SUCRA = 79.9), followed by CWI (SUCRA = 71.5) and CWT (SUCRA = 51.8), with WBC lowest (SUCRA = 22.5).
- 72 h: CWI highest, followed by LCT, with CON lowest.

These results suggest that CWI is most effective in the early recovery phase (1–24 h), whereas LCT shows greater efficacy in later recovery (48 h), and WBC may have limited impact on DOMS reduction.

##### 3.4.4.3 Cmj

The network geometry for CMJ is shown in Figure 3(t–x). Cold-water immersion (CWI) and contrast water therapy (CWT) were the most frequently applied interventions, while localized cryotherapy (LCT) was applied least often. At the immediate and 1 h time points, no LCT intervention arms were available, and all comparisons were based on mixed evidence. At 24 h, 48 h, and 72 h, all comparisons except those involving LCT were based on mixed evidence, while LCT comparisons were indirect. Network consistency was assessed using loop inconsistency tests, consistency models, and node-splitting analyses. Loop inconsistency tests indicated that all triangular loops involving WBC, CWI, CWT, and control (CON) were consistent (*P* > 0.05). LCT comparisons were only indirect and did not form closed loops, while other loops demonstrated good consistency. Inconsistency model testing yielded P-values > 0.05 for all outcomes, indicating no significant inconsistency and supporting the use of the consistency model. Node segmentation method showed that no significant statistical inconsistency was found between direct comparison and indirect comparison of each outcome index within the existing evidence network (*P* > 0.05). However, considering the sparse evidence of LCT and the lack of direct comparison, this result is not absolutely reliable. Therefore, CMJ result analysis should be interpreted with caution in combination with network structure characteristics.Funnel plot assessments demonstrated good symmetry, indicating minimal influence of publication bias or small-study effects (Appendix, Table e; Appendix, Figures e).

##### 3.4.4.4 Pooled effect estimates and sucra rankings

Overall, different cryotherapy modalities showed varying effectiveness in enhancing CMJ performance compared with control across multiple post-exercise time points. Detailed analysis revealed the following: (Figures 4e, 5e)

- Immediate: WBC (MD = 5.52, 95% CI (−1.58, 12.62), *P* > 0.05), CWI (MD = 1.01, 95% CI (−4.28, 6.30), *P* > 0.05), and CWT (MD = 1.52, 95% CI (−5.84, 8.88), *P* > 0.05) showed some improvement in CMJ, but results were not statistically significant.
- 1 h: WBC (MD = 9.15, 95% CI (5.12, 13.17), *P* < 0.05) demonstrated the most significant improvement, with statistical significance.
- 24 h: WBC (MD = 10.79, 95% CI (1.16, 20.42), *P* < 0.05) and CWI (MD = 4.81, 95% CI (0.88, 8.75), *P* < 0.05) showed significant effects.
- 48 h: WBC[MD = 10.50,95% (3.37,17.62), *P* < 0.05], CWT[MD = 7.10,95% (0.12,14.07), *P* < 0.05], the effect was significant and statistically significant.
- 72 h: WBC[MD = 8.92,95%(-0.61,18.45), *P* = 0.066] and CWI[MD = 5.23,95% (−0.27,10.73), *P* = 0.063] had an upward trend in improving the effect of CMJ, but did not reach statistical significance.

SUCRA Rankings for CMJ, noting that rankings are based on reducing performance loss (i.e., higher SUCRA indicates better performance retention), were interpreted as follows:

- Immediate and 1 h: WBC had the highest probability of being the most effective intervention, followed by CWT and CWI, with CON lowest.
- 24 h, 48 h, and 72 h: WBC had the highest probability of being the most effective, followed by LCT and CWI, with CON lowest.

These results indicate that WBC consistently provides the greatest enhancement of CMJ performance across all post-exercise time points, while CWI and other modalities show time-dependent improvements.

## 4 Discussion

This study, through a systematic review and network meta-analysis (NMA), is the first to comprehensively compare the effects of four common cryotherapy modalities on post-exercise subjective recovery, physical performance, and inflammatory markers across multiple time points. The findings can be interpreted in several key aspects. First, the effects of different cryotherapy modalities on recovery outcomes vary considerably across time, reflecting the highly time-dependent nature of post-exercise physiological recovery (4). Overall, cryotherapy interventions show limited efficacy in the immediate and early phases (immediate–1 h) post-exercise, with effects not reaching statistical significance. In contrast, moderate-to-late phases (24–72 h) demonstrate significant reductions in muscle soreness and inflammatory markers, consistent with the temporal pathophysiology of delayed-onset muscle soreness (DOMS). Second, regarding inflammatory biomarkers, WBC exhibited the most pronounced reduction in CK at 48 h and 72 h, whereas CWI ranked highest for IL-6 suppression in the immediate, 1 h, and 24 h periods, suggesting that different cryotherapy modalities may modulate exercise-induced inflammation through distinct physiological mechanisms. Third, in terms of recovery of physical performance, although cryotherapy did not significantly enhance CMJ immediately post-exercise, WBC significantly improved CMJ performance at 1 h, 24 h, and 48 h, indicating potential advantages in restoring neuromuscular function and explosive power. Based on the current sparse and uneven evidence network, our analysis results show that there is no “universal cooling therapy” that is dominant at all time points and on all outcome measures. It is important to emphasize that for interventions such as LCT, the ranking and effect size estimation are highly dependent on indirect comparisons, so the relevant conclusions are more uncertain. More direct comparative studies are needed to verify this in the future.

Eccentric exercise, such as high-intensity interval training and resistance training, can easily lead to mechanical damage to muscle fiber microstructure, causing significant local inflammation and CK leakage. CWI is effective in limiting vascular permeability and inflammatory cell infiltration in the injured area by rapid local deep cooling, which may make it particularly effective in dealing with this early inflammatory response dominated by mechanical damage. The extremely low temperature systemic stimulation of WBC may more strongly activate the sympathetic nervous system and hypothalamic-pituitary-adrenal axis, and promote the release of hormones with systemic anti-inflammatory and excitatory effects such as norepinephrine. Such systemic modulation may take longer to manifest but may be more beneficial in promoting overall recovery of neuromuscular function and mitigating later declines in performance caused by central fatigue and systemic inflammatory responses. In addition, for endurance exercise with metabolic stress as the main focus, cooling therapy may promote recovery mainly by reducing core temperature and cardiovascular stress, but its effect pattern on DOMS and inflammatory factors may be different from that of the eccentric exercise model (4).

Exercise-induced muscle damage is typically accompanied by disruption of muscle membrane integrity and a cascade of inflammatory responses, resulting in elevated circulating CK, IL-6, and CRP levels (54). Our findings indicate that cryotherapy has limited modulatory effects on CK and CRP in the immediate phase but demonstrates more pronounced effects at 48–72 h, particularly with WBC. This may be related to cryotherapy's suppression of secondary muscle damage. By lowering tissue temperature, cryotherapy induces vasoconstriction, reduces local blood flow and metabolic rate, thereby limiting migration of inflammatory cells and release of pro-inflammatory mediators (60). WBC, due to its extremely low temperature (−110 ℃ to −140 ℃) and whole-body exposure, may provide stronger systemic inflammatory and central nervous system stimuli, explaining its superior efficacy on CK and CRP at later phases (61). In contrast, the early IL-6 suppression observed with CWI may reflect rapid reductions in local tissue temperature and acute modulation of early inflammatory signaling. IL-6, a cytokine with both pro- and anti-inflammatory properties, rises rapidly during early post-exercise periods and is highly sensitive to cold stimuli (10), explaining the early-phase advantage of CWI. DOMS typically peaks 24–72 h post-exercise, associated with microstructural muscle fiber damage, inflammatory responses, and increased mechanical nociceptive sensitivity (2). Cryotherapy did not significantly alleviate DOMS immediately post-exercise, but CWI and LCT were most effective at 24 h and 48 h. Notably, WBC consistently showed benefits in CMJ recovery during 1–48 h, possibly due to its effects on autonomic nervous system balance and central fatigue recovery. Evidence suggests that cold exposure can modulate sympathetic-parasympathetic activity and improve subjective recovery perception, thereby enhancing performance recovery (2, 10, 61).

Previous systematic reviews have mostly focused on single cryotherapy modalities or single time points and relied on conventional pairwise meta-analysis (62). Methodologically, this study employed NMA to enable direct and indirect comparisons among multiple cryotherapy modalities and introduced a multi-time-point framework to avoid interpretive bias caused by combining outcomes across different physiological phases. By integrating subjective, functional, and biochemical outcomes, the clinical applicability of the findings is enhanced.

This study has several limitations. First, the limited efficacy of cryotherapy in the early phase may reflect the influence of different early post-exercise physiological mechanisms and neuromuscular function decline, compounded by the small number of studies and limited sample sizes at immediate and 1 h time points. Second, heterogeneity exists within the same intervention modality: included WBC protocols ranged from −30 ℃ to −135 ℃, and CWI ranged from 5 ℃ to 15 ℃, with intervention durations from 1.5 to 20 minutes, introducing potential variability in outcomes. Third, the studies did not examine the influence of sex, participant type, training level, or exercise intensity on cryotherapy response. Fourth, the validity of a network meta-analysis rests on the transitivity assumption that different sets of studies used for indirect comparisons are considered comparable in key characteristics. The cooling interventions included in this study had differences in specific parameters of temperature, duration and frequency, and the number of studies and population characteristics of different intervention methods were unevenly distributed. Although we found no significant inconsistency in the statistical analysis, the structural imbalance of the evidence network may potentially affect the validity of the transitivity hypothesis, such as CWI and CON with abundant evidence while LCT and other cooling modalities are only indirectly connected. Ideally, the influence of these effect modifiers would be explored by subgroup analysis or meta-regression, but we were not able to perform such analyses because of the limited number of studies within each comparison group. This constitutes an important limitation of the study and means that results from comparisons of different cooling modalities, especially those that rely heavily on circumstantial evidence, should be viewed as best estimates within the available evidence network, rather than firm conclusions.Fifth, the inverse jump (CMJ) used in this study is a valid objective indicator to assess neuromuscular function and burst power, but it mainly reflects the peripheral functional state of lower limb muscles, has limited capture of central fatigue, and pain perception may be significantly affected by individual tolerance, psychological expectations, and placebo effects. DOMS itself is a kind of multi-dimensional subjective feeling, usually using visual analogue scale (VAS) to evaluate, but it and objective muscle damage or function decline does not completely have linear effect relationship, in the assessment of delayed onset muscle soreness (DOMS) and fatigue, the index combination used in this study is still complex.

Based on the results of the timing analysis of this study, we propose more practical recommendations: (1) If the primary goal after exercise is rapid pain relief, CWI should be preferentially used within 1 hour after exercise (e.g., 10-15°C immersion for 10-15 minutes). If the goal is to promote recovery of athletic performance during subsequent training or competition sessions, particularly after 24 to 72 hours, WBC may provide more sustained benefits. (2) For athletes mainly engaged in eccentric strength training, attention should be paid to the role of CWI in alleviating early muscle injury; However, for team ball players with an intensive schedule who need to quickly recover neuromuscular function, WBC can be considered as part of the routine recovery process. (3) Given the complementary mechanisms of different cooling therapies, sequential application can be explored in practice, such as CWI immediately after exercise to control acute inflammation, followed by WBC 24 hours later to promote systemic recovery. It must be emphasized that any cooling intervention should be performed under professional guidance and strictly exclude contraindications such as cardiovascular disease.

Although this study has made certain findings, the following directions are worthy of further exploration in the future: (1) The deepening of mechanism research: in the future, the specific effects of different cooling therapies on central and peripheral fatigue and pain perception pathways should be directly verified in human experiments by combining molecular biological techniques such as detecting the levels of proteins and hormones in specific inflammatory pathways, and using neurophysiological means. (2) Establishment of individualized regimen: research should pay attention to individual differences and the optimal combination of cooling parameters. To explore whether there is a “dose-response” relationship specific to different populations and types of exercise. (3) Long-term effects and safety: the current evidence mostly focuses on single or short-term effects. In the future, high-quality long-term follow-up studies are needed to evaluate the cumulative effects of regular cooling therapy on exercise adaptation, immune function, and potential risks. (4) Integration of multimodal recovery strategies: cooling therapy should not be viewed in isolation. Future studies should investigate the synergistic effects of cooling therapy with other recovery methods such as nutritional supplements such as protein, antioxidants, compression equipment, and sleep management to construct an optimal comprehensive recovery program.

## 5 Conclusion

The findings of this systematic review and network meta-analysis indicate that cryotherapy is an effective intervention for promoting recovery following acute exercise; however, its efficacy is highly dependent on the type of intervention and the timing of application. Different cryotherapy modalities exhibit varying advantages across DOMS, performance outcomes, and inflammatory markers, highlighting the importance of personalized, time- and goal-oriented cryotherapy strategies in post-exercise recovery practice. This study provides athletes, coaches, and clinical practitioners with a systematic and reliable evidence-based framework to guide recovery decision-making following high-intensity training and competition.

## Data Availability

The datasets presented in this study can be found in online repositories. The names of the repository/repositories and accession number(s) can be found in the article/Supplementary Material.
